# Why patient organizations are important to improve care for people with Lynch syndrome

**DOI:** 10.1007/s10689-026-00572-1

**Published:** 2026-05-20

**Authors:** Jurgen Seppen

**Affiliations:** 1 Tytgat Institute for Liver and Intestinal Research, Meibergdreef 69, 1105BK, Amsterdam UMC Location University of Amsterdam, Amsterdam, The Netherlands; 2 Stichting Lynch-Polyposis, p/a NFK, Domus Medica, Mercatorlaan 1200, 3528 BL Utrecht, The Netherlands

**Keywords:** Lynch syndrome, Shared decision making, Patient advocacy

## Abstract

Improving health care requires patient involvement. For people with Lynch syndrome, general and specific areas can be identified in which it is important for health care professionals to collaborate with patients to improve care. This collaboration is mutually beneficial; better care for the patients, reduction of work load and higher chance of funding of research proposals for health care professionals. Patient organizations are comprised of patients that are often more educated and motivated to participate in discussions and workshops. In addition, patient organizations have access to a network of patients that can provide a broad overview of the needs and requirements of this group. Thus, patient organizations are the partners of choice in the collaboration of health care providers and patients. In this commentary, I outline the areas in which physicians and patients should collaborate; Providing information, research and clinical trial design, and psychological care and peer support.

## Introduction

Lynch syndrome is not a rare disorder, a recent paper estimates that up to 1 in 100 people may have a pathogenic mutation in one of the Lynch syndrome genes [1].

Most general practitioners will therefore have several people with Lynch syndrome in their practice and people with Lynch syndrome will commmonly be seen in Gastroenterology, Gynaecology and Clinical genetics departments. Given the large amount of people with Lynch syndrome, it is important to give an overview of areas where patients and health care professionals should collaborate to improve care.

A study in patients with Lynch syndrome reported that medical information about Lynch syndrome and psychological care for Lynch syndrome patients is not optimal. In addition, this study also identified that the knowledge of health care providers about Lynch syndrome needs improvement [2].

Patient participation in health care encompasses a wide variety of areas and is generally considered to be positive both for patients and health care professionals [3].

Patient organizations can play an important role in the collaboration between care providers and patients because they are usually comprised of motivated people that have access to a broad support base of other patients. This will ascertain a nuanced and constructive role of patients in improving healthcare [4].

Together, these considerations are a strong indication that there is a need for health care providers and patients to collaborate to improve care.

For Lynch syndrome it is possible to identify different aspects of care where the input of patients and patient organizations is essential to improve healthcare. In this commentary I summarize the most important aspects where care providers and patients should collaborate and provide some recommendations.

## Providing accurate information

The “Stichting Lynch-Polyposis” is a Dutch patient organisation, representing the interests of people with Lynch syndrome and various forms of Polyposis. In a survey from 2024 among donors of the “Stichting Lynch-polyposis” 83% of 111 respondents indicated their reason for their support was the need for more information. In 2020 in a similar survey, 85% of 113 respondents indicated the main reason they participate in activities organized by the “stichting Lynch-Polyposis” is the need for information.

These resultsare not surprising since there is a large gap in knowledge between physicians and patients that is difficult to bridge in the consultation room. The information needs of Lynch syndrome patients are covered by different specialisms. Some examples include; colonoscopy screening, the procedures and required intervals. Determining carrier status, identifying and informing potentially affected family members. Conceiving children, possibility of preimplantation screening. Psychological help. Decisions about (preventive) surgery.

### Generation of information materials

Patient organizations should be involved in generating information materials because they have a good overview about the information needs of people with Lynch syndrome. This process is also known as co-creation [5] Some of the best practices identified in the co-creation process are involving patients and healthcare professionals from the beginning and empower patients to have the final say in the created materials [5].

### Flipping the consultation room

It is well established that orally provided information is not very well retained. This has been studied in a consult setting with test subjects comprised of students [6]. In a consultation room where the patient may possibly be anxious and under time constraint, information transfer is likely to be even less effective than with students.

A potentially effective way to better inform patients is to provide them with information prior to their visit to the clinic. This is a strategy that in education is called a “flipped classroom” where students are provided with the lecture information before they enter their classes. This approach has been shown to be superior in uptake of information to traditional forms of teaching [7]. A similar strategy can be used to inform patients before their visit to the clinic [8, 9]. By providing patients with information prior to their visit to the clinic, the amount of questions will be reduced and more focused. This is mutually beneficial as it will lead to better informed patients while at the same time reducing the time needed to inform patients thus reducing the workload for clinicians as well.

### Patients with lower health literacy

The heterogeneity of patients makes it difficult to come up with general recommendations about how to construct information materials that can bridge the information gap between patients and health care professionals. It is estimated that around 25% of people has limited health literacy [10].

It is this group that is most vulnerable and difficult to reach. Efforts to improve communication should therefore be addressed to this part of the patient population. Communication to lay people is a specialized field, it is advisable to involve communication professionals in writing brochures or online documents. Information tailored to children should also be provided and requires the input of children’s health care professionals. However, it remains crucial that these initiatives are discussed and tested with patient groups. Patient organizations provide the platforms and networks that are necessary to evaluate the effectiveness of these communications.

### Informing first line care providers

Providing information to patients is important in specialist care but perhaps more important in the first line [11]. There is a knowledge gap between specialist physicians and general practitioners about Lynch syndrome and its consequences. Since general practitioners are often the first source of information for family members it is imperative they can find reliable information as well [11]. Anecdotal evidence from patients in the Netherlands indicates that unreliable information about negative financial consequences of genetic testing is still being disseminated, this may induce unnecessary fears and lead to reluctance in uptake of testing by potentially affected people.

### Online sources of information

Patients need guidance to traverse the vast amount of online information that is available. The access to online health information can improve care but only when there is a good relation between the patients and care provider and the obtained (mis)information can be discussed openly [12]. There are many reliable sources of information and patients need to be referred to those. It is therefore important that patients feel free to discuss information they found online with their care givers [13]. This may increase trust and help patients find reliable sources of information.

### Use of generative AI as information source

The rapid development of generative AI has also led to the development of tools for patient diagnosis and shared decision making. While these tools hold great promise in improving care and diagnosis [14], improvement in trust and implementation are still required [15]. It is likely that AI tools will transform the field of clinical genetics and have a great potential to help patients better inform themselves and family members about their disorder [16].

At this moment there are many different AI tools available that patients can query. However, the reliability of answers that generative AI models provide is uncertain and, as with all forms of online information, close monitoring of information that patients obtain is necessary. Studies in gynaecology and cardiology indicate that generative AI models perform well in informing patients but verification of the information with an expert remains necessary [17, 18].

Here patient organizations and health care professionals must collaborate to prevent patients from obtaining false information and, when required, correct false information and direct patients to reliable information sources.

### Finding undiagnosed patients with Lynch syndrome

Given the high prevalence of Lynch syndrome it is striking that Lynch syndrome is severely underdiagnosed, many people with Lynch syndrome are not aware they have this disorder. Some estimates put the percentage of undiagnosed Lynch syndrome patients at 95% [19].

By collaboration with patient organizations, health care providers can develop information material about Lynch syndrome that will help patients inform their family members and motivate them to get DNA testing and proper surveillance. The benefits and potential to increase life expectancy [20] must be outlined clearly. This information material can also be used when family members consult with their general practitioner to discuss whether DNA testing is necessary for them.

It is also important that potential fears, for instance about negative consequences in obtaining loans, must be addressed. This obviously requires knowledge about relevant legislation that can differ per country. Peer support through patient organizations can provide an important role in this process because it can provide personal experiences about informing family members. Indeed, in people with inherited breast cancer disorders due to BRCA1/2 deficiency, this approach has been shown to be successful [21].

### Surveillance and management

In our surveys and communication with patients (surveys Stichting Lynch-polyposis), the regular colonoscopy surveillance is seen as one of the biggest burdens for patients with Lynch syndrome. Communication about the need of this surveillance is essential. At the same time, flexibility for the patient in determining the interval that suits them best will increase participation. Here, shared decision making and flexibility in implementing nationally established intervals for colonoscopy is essential.

Prophylactic hysterectomy and bilateral salpingo-oophorectomy can be advised due to the high lifetime risk of endometrial and ovarian cancer in women with Lynch syndrome. In addition, preventive colorectal surgery may be considered in selected patients at very high risk of colorectal cancer [22]. Preventive surgery should always be discussed with the patients in a careful manner. Shared decision making is of the greatest importance in this area [23].

Patients need to be informed that prophylactic gynaecological surgery makes natural conception impossible, so these procedures are usually performed after completion of childbearing. Additionally, they should understand the bowel function changes (e.g., increased frequency and loose stools) that may follow extensive colorectal resection.. Here, patient organizations can play an important role by providing peer support about difficult decisions and connect patients to peers that have struggled with the same questions. At the same time, physicians should always keep in mind that patient empowerment is important to those patients who want an active role in their treatment or screening, while others may prefer to largely rely on the advice of care providers.

## Research

Patient organizations are the ideal partners to collaborate with when patient involvement is required in research because they usually have a diverse composition and are therefore familiar with different aspects of Lynch syndrome for which research is needed to improve treatment, diagnosis or quality of life. Patients involvement in clinical trial design has been shown to benefit both patients and clinicians [24, 25].

Many funding organizations already require patient involvement as part of research proposals. However, because patient involvement is beneficial for both research and patients, it should be mandatory for researchers to search for patient input when drafting new proposals. To obtain relevant feedback it is necessary that researchers provide patients with a summary that is comprehensible to non-experts [26]. Overinflating the relevance of the research should be avoided. Patients understand the need for basic biological research but will need information directed towards lay people to assess the quality of the proposals in this area. To facilitate recruitment of patients in clinical trials, collaboration with patient organizations is necessary. They can help in pinpointing elements of the study, such as screening intervals, visits to the clinic and invasive procedures, that are potentially burdensome. Collaboration with patient organizations will ensure the most optimal trial design for patient enrollment. In addition, when patient organizations are “on board” and part of the study design, they can help to reach out to their network of patients to recruit participants.

In basic biological science, patient involvement is more limited than in the clinical area. However, there is much to gain for both patient and researchers in collaborating in basic science as well [27]. A better understanding of the role of basic science in the developments of new treatments and diagnostics is important for patients. At the same time, a better understating of basic scientists about the needs and problems of patients can be very motivating for researchers and improve research [28].

Most patient organizations rely on volunteers that do this work in their spare time. Preparing an advice and or search for patients that can give well informed advice takes time. Last minute request for participation should therefore be avoided. Patient organizations are often comprised of volunteers and have, especially when relying solely on membership contributions, limited financial means. When research proposals are developed in partnership with patient organizations, a portion of the funding should be allocated to cover expenses for the patients who contribute to study design and trial oversightThe “Stichting Lynch Polyposis” receives complaints from patients that they did not receive feedback about the results of the trial or other type of research they participated in. It is important to inform patients of the results of clinical trials and research, even if these results are preliminary or inconclusive. Patients understand better what their role in the research was and will be better motivated for future collaborations.

## Peer support

Although most hospitals will have specialists available to provide psychological help for patients, it is important for many patients to be able to talk to people with the same condition. Peer support is different from professional support by hospital staff, and both types of support should be seen as complementary [29].

Patient organizations often have a network of peer groups that are experienced in talking to fellow patients and can provide support that is impossible to get from a hospital psychologist. It is therefore important that physicians are able to refer patients to peer groups.

Research evaluating the cooperation of genetic councilors and peer support indicates that, although most patient support organizations that have relationships with genetic counselors report satisfaction, barriers such as limited funding and access remain. Addressing these could further strengthen collaboration [30].

In addition to helping patients cope with Lynch syndrome, peer support is also suggested to help finding affected family members by increasing uptake of genetic testing [21, 31].

Online peer support is easily accessible for people with Lynch syndrome and these platforms fulfill a great need. However, people with hereditary cancer also indicate that they appreciate involvement of medical experts in these platforms [32].

This is especially important because care must be taken that no misinformation is spread in these online communities. Patient organizations can fulfill these needs by providing “quality control” because their members can both act as peers and experts by obtaining information from their network of physicians.

## Recommendations and conclusions

### Recommendations

Physicians should refer patients to patients organizations when possible and keep close contact with representatives of these organizations. However, patient organizations receive many medical questions from patients about their disorder they cannot and should not answer. Patients organizations should always refrain from giving medical advice. Instead they need a platform of clinicians to which they can refer these questions to, while at the same time ensuring that the clinicians remain anonymous for the person asking the question. These aspects require good collaboration and contacts between patient organizations and clinicians.

Besides the recommendations outlined above, a practical way in which the goal of collaboration between patients organizations and physicians can be strengthened is by organizing symposia.

The Dutch Lynch Polyposis patient organization (Stichting Lynch Polyposis) organizes a yearly meeting that usually draws an audience of 120–150 people with Lynch syndrome and polyposis. There are expert speakers that highlight recent research and treatment developments or more general topics such as colonoscopy preparation, lifestyle and genetic screening. In addition to the general meeting for all Dutch patients, several medical centers in the Netherlands organize meetings aimed at local patients that have a similar setup. These local meetings are also supported by the patient organization, either with advice about topics or practical help in organization. Topics for presentations at all these meetings are always discussed with patient representatives and based on feedback from the network of patients the organization has access to.

In addition, these meeting can also be used to announce the results of research and future research projects and can be used to get direct feedback from patients about study design and goals.

Because patients are in a group of peers, they often feel free to ask questions that they may still have after a consultation or that they were reluctant to ask. It is important when organizing these meetings to allow time for the participants to meet peers and discuss topics relevant for the patient group. It is also valuable, especially for young physicians and researchers, to meet patients in a more informal setting.

Organizing patients meetings rely on the participation of physicians, researchers, patient volunteers and attending patients. The feedback our organization gets from these meetings is overwhelmingly positive, both from the audience and organizing side.

Many of the goals outlined in this review, information, research design and peer support can be addressed at these meetings. Meetings like this should therefore be considered as a best practice that greatly benefits both patients and care providers.

### Conclusions

Patient organizations play an indispensable role in improving care and well being for people with Lynch syndrome. Physicians and patient organizations should collaborate closely to obtain the most advantage of this relationship. While there are many benefits in patient participation [33], it also clear that hurdles remain and a continuous effort to improve this collaboration needs to be made [34].

## Data Availability

No datasets were generated or analysed during the current study.
